# 8-Chloro­methyl-5-(2,5-dioxooxolan-3-yl)-3,3a,4,5-tetra­hydro-1*H*-naphtho­[1,2-*c*]furan-1,3-dione

**DOI:** 10.1107/S1600536813014943

**Published:** 2013-06-08

**Authors:** Y. Z. Guo, Y. Z. Song, J. G. Liu, S. Y. Yang

**Affiliations:** a Laboratory of Advanced Polymer Materials, Institute of Chemistry, Chinese Academy of Sciences (ICCAS), Beijing 100190, People’s Republic of China; b Beijing BOE Display Technology Co., Ltd, No. 118 Jinghaiyilu, BDA, Beijing 100176

## Abstract

The title compound, C_17_H_13_ClO_6_, is an asymmetric alicyclic dianhydride containing a chloro­methyl-substituted tetra­hydro­naphthalene moiety. The cyclo­hexene ring in the tetra­hydro­naphthalene moiety exhibits an envelope conformation with the tertiary C atom as the flap The dihedral angle between the two anhydride rings is 79.96 (6)°, while those between the benzene ring and the non-fused and fused anhydride rings are 71.03 (5) and 42.57 (7)°, respectively. In the crystal, mol­ecules are connected by weak C—H⋯O inter­actions, forming a three-dimensional supramolecular structure.

## Related literature

For background to polyimides, see: Li *et al.* (2005 ▶); Liaw *et al.* (2012 ▶); Zhang *et al.* (2003 ▶); Zhong *et al.* (2004 ▶). For background to and applications of tetra­hydro­naphthalene-containing alicyclic dianhydrides, see: Guo, Shen *et al.* (2013 ▶). For the structure of a related compound, see: Guo, Liu & Yang (2013 ▶) and for its synthesis, see: Hall *et al.* (1982 ▶); Guo *et al.* (2012 ▶). For puckering parameters, see: Cremer & Pople (1975 ▶).
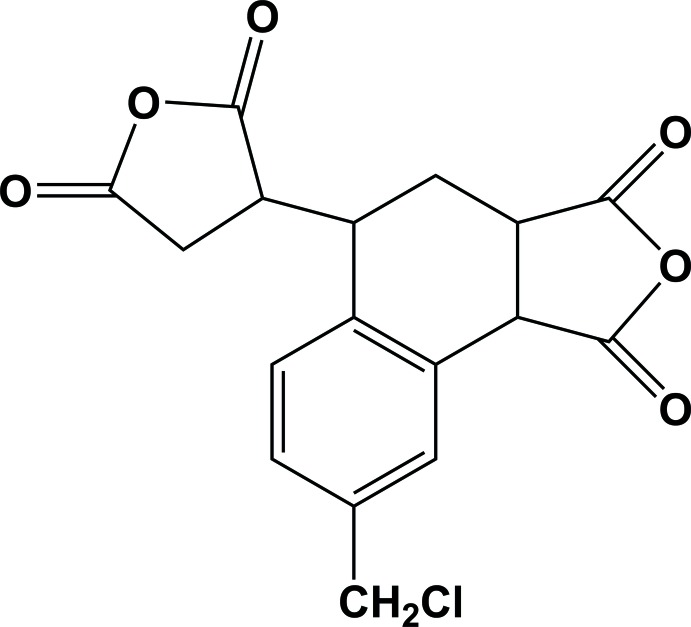


## Experimental

### 

#### Crystal data

C_17_H_13_ClO_6_
*M* *_r_* = 348.72
Triclinic, 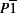

*a* = 6.8988 (15) Å
*b* = 9.140 (2) Å
*c* = 11.950 (3) Å
α = 80.937 (9)°
β = 75.365 (8)°
γ = 79.614 (8)°
*V* = 712.1 (3) Å^3^
*Z* = 2
Mo *K*α radiation
μ = 0.30 mm^−1^
*T* = 173 K
0.41 × 0.21 × 0.15 mm

#### Data collection

Rigaku Saturn724+ CCD diffractometer
Absorption correction: multi-scan (*CrystalClear*; Rigaku, 2008) ▶ *T*_min_ = 0.702, *T*_max_ = 1.000
9192 measured reflections
3238 independent reflections
3034 reflections with *I* > 2σ(*I*)
*R*_int_ = 0.035

#### Refinement

*R*[*F* ^2^ > 2σ(*F* ^2^)] = 0.042
*wR*(*F* ^2^) = 0.108
*S* = 1.10
3238 reflections
217 parameters
H-atom parameters constrained
Δρ_max_ = 0.38 e Å^−3^
Δρ_min_ = −0.44 e Å^−3^

### 

Data collection: *CrystalClear* (Rigaku, 2008) ▶; cell refinement: *CrystalClear*; data reduction: *CrystalClear*; program(s) used to solve structure: *SHELXS97* (Sheldrick, 2008 ▶); program(s) used to refine structure: *SHELXL97* (Sheldrick, 2008 ▶); molecular graphics: *OLEX2* (Dolomanov *et al.*, 2009 ▶); software used to prepare material for publication: *OLEX2*.

## Supplementary Material

Crystal structure: contains datablock(s) I, global. DOI: 10.1107/S1600536813014943/zp2004sup1.cif

Structure factors: contains datablock(s) I. DOI: 10.1107/S1600536813014943/zp2004Isup2.hkl

Supplementary material file. DOI: 10.1107/S1600536813014943/zp2004Isup3.cml

Additional supplementary materials:  crystallographic information; 3D view; checkCIF report

## Figures and Tables

**Table 1:** Hydrogen-bond geometry (Å, °)

*D*—H⋯*A*	*D*—H	H⋯*A*	*D*⋯*A*	*D*—H⋯*A*
C3—H3*B*⋯O4^i^	0.99	2.51	3.420 (2)	153
C5—H5⋯O1^ii^	1.00	2.59	3.386 (2)	136
C7—H7⋯O4^i^	1.00	2.51	3.468 (2)	160

Symmetry codes: (i) 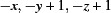; (ii) 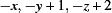.

**Fig. 1.**
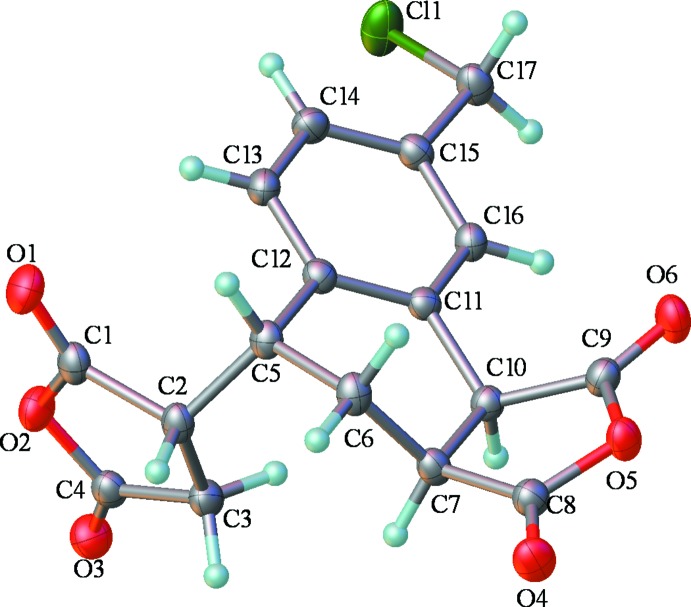
The molecular structure of the title compound, showing diaplacement ellipsoids at the 30% probability level.

## supplementary crystallographic information

### Comment

Polyimide (PI) is an important class of high performance polymers in the current industry. Functional PI materials have been widely used in microelectronic, optoelectronic and advanced display areas (Liaw *et al.*, 2012). Chloromethyl is an important active species for functionalization of PI materials (Li *et al.*, 2005). For instance, PIs which are highly sensitive to ultraviolet lights of high-pressure mercury lamps have been successfully developed *via* the reaction of chloromethyl substituted in the PI molecules with photosensitive substances, such as cinnamic acid (Zhang *et al.*, 2003). In addition, chloromethyl-containing PIs exhibited good sensitivity to linearly polarized ultraviolet light, which making it possible using the PIs for the photoalignment fabrication of liquid crystal molecules in advanced liquid crystal display devices (Zhong *et al.*, 2004). In the current work, we reported a novel chloromethyl-containing alicyclic dianhydride monomer. The molecular structure of the title compound is shown in Fig. 1. The compound has an asymmetrical structure and the dihedral angle between the two anhydride rings is 79.96 (6)° while the dihedral angles between the benzene ring and the anhydride ring 1(C1—C2—C3—C4—O2) and anhydride ring 2 (C7—C8—C9—C10—O5) are 71.03 (5)° and 42.57 (7)°, respectively. The six-membered cyclohexene ring in the tetra-hydronaphthalene residue exhibits an envelope conformation with puckering parameters of Q = 0.4805 (17) Å, θ=57.46 (19)° and φ =58.9 (2)°. =122.8 (2)° and φ=300.7 (2)° (Cremer & Pople, 1975). There is an intramolecular C—H···Cl hydrogen bond in the molecule, while in the crystal, molecules are connected by week C—H···O intermolecular interactions, as shown in Table 1.

### Experimental

Into a 500 ml three-necked flask equipped with a mechanical stirrer, a nitric oxide inlet, and a condenser, 43.75 g(0.446 mol) of maleic anhydride, 104.09 g(0.682 mol) of 4-chloromethylstyrene, 0.1138 g(0.5 mmol) of 2,5-di-*tert*-butyl hydroquinone were added. The reaction mixture was heated to 120°C and maintained for 6 h under nitric oxide. The produced red-brown nitrogen oxide gas was trapped by passing through an aqueous solution of 20 wt% sodium hydroxide. White needle crystals were formed. After the reaction was completed, 60 ml of acetonitrile was added and the solution was refluxed for about 0.5 h. Then 60 ml of toluene was added and the reactino mixture was cooled to room temperature. The produced white needle crystals was collected by filtration and the solid was washed with toluene and petroleum ether in succession. After being dried in vacuum, the pure MCTDA was obtained as white crystals. Yield: 59.49 g(76.5%). Elemental analysis: calculated for C_17_H_13_ClO_6_:*C*,58.55; H:3.76%. Found: C:58.71; H:3.85%. EI—MS, m/*z*:142(*M*^+^-176, 100%). Colourless single crystals were grown by slow evaporation of an acetonitrile solution over a period of several days.

### Refinement

All H atoms were positioned geometrically (C-H=0.95-1.00 Å) and refined using a riding model with the *U*_iso_(H)=1.2 *U*_eq_C for both of the aromatic ring and aliphatic chain.

### Crystal data

**Table:**

C_17_H_13_ClO_6_	*Z* = 2
*M**_r_* = 348.72	*F*(000) = 360
Triclinic, *P*1	*D*_x_ = 1.626 Mg m^−^^3^
Hall symbol: -p 1	Melting point: 502 K
*a* = 6.8988 (15) Å	Mo *K*α radiation, λ = 0.71073 Å
*b* = 9.140 (2) Å	Cell parameters from 2542 reflections
*c* = 11.950 (3) Å	θ = 2.3–27.5°
α = 80.937 (9)°	µ = 0.30 mm^−^^1^
β = 75.365 (8)°	*T* = 173 K
γ = 79.614 (8)°	Block, colourless
*V* = 712.1 (3) Å^3^	0.41 × 0.21 × 0.15 mm

### Data collection

**Table:**

Rigaku Saturn724+ CCD diffractometer	3238 independent reflections
Radiation source: sealed tube	3034 reflections with *I* > 2σ(*I*)
Graphite monochromator	*R*_int_ = 0.035
ω scans at fixed χ = 45°	θ_max_ = 27.5°, θ_min_ = 3.1°
Absorption correction: multi-scan (*CrystalClear*; Rigaku, 2008)	*h* = −8→8
*T*_min_ = 0.702, *T*_max_ = 1.000	*k* = −11→11
9192 measured reflections	*l* = −15→15

### Refinement

**Table:**

Refinement on *F*^2^	Primary atom site location: structure-invariant direct methods
Least-squares matrix: full	Secondary atom site location: difference Fourier map
*R*[*F*^2^ > 2σ(*F*^2^)] = 0.042	Hydrogen site location: inferred from neighbouring sites
*wR*(*F*^2^) = 0.108	H-atom parameters constrained
*S* = 1.10	*w* = 1/[σ^2^(*F*_o_^2^) + (0.0492*P*)^2^ + 0.3488*P*] where *P* = (*F*_o_^2^ + 2*F*_c_^2^)/3
3238 reflections	(Δ/σ)_max_ = 0.001
217 parameters	Δρ_max_ = 0.38 e Å^−^^3^
0 restraints	Δρ_min_ = −0.44 e Å^−^^3^

### Special details

**Table:**

Geometry. All e.s.d.'s (except the e.s.d. in the dihedral angle between two l.s. planes) are estimated using the full covariance matrix. The cell e.s.d.'s are taken into account individually in the estimation of e.s.d.'s in distances, angles and torsion angles; correlations between e.s.d.'s in cell parameters are only used when they are defined by crystal symmetry. An approximate (isotropic) treatment of cell e.s.d.'s is used for estimating e.s.d.'s involving l.s. planes.
Refinement. Refinement of *F*^2^ against ALL reflections. The weighted *R*-factor *wR* and goodness of fit *S* are based on *F*^2^, conventional *R*-factors *R* are based on *F*, with *F* set to zero for negative *F*^2^. The threshold expression of *F*^2^ > σ(*F*^2^) is used only for calculating *R*-factors(gt) *etc*. and is not relevant to the choice of reflections for refinement. *R*-factors based on *F*^2^ are statistically about twice as large as those based on *F*, and *R*- factors based on ALL data will be even larger.

### Fractional atomic coordinates and isotropic or equivalent isotropic displacement parameters (Å^2^)

**Table:**

	*x*	*y*	*z*	*U*_iso_*/*U*_eq_	
Cl1	1.05131 (7)	−0.07996 (5)	0.86631 (4)	0.03800 (15)	
O1	0.26974 (19)	0.57365 (14)	0.98000 (10)	0.0314 (3)	
O2	0.50528 (18)	0.64807 (13)	0.82479 (10)	0.0277 (3)	
O3	0.70203 (18)	0.68985 (14)	0.64654 (11)	0.0303 (3)	
O4	−0.16049 (18)	0.33284 (13)	0.54844 (11)	0.0301 (3)	
O5	0.05290 (17)	0.11884 (12)	0.56476 (10)	0.0258 (3)	
O6	0.31175 (19)	−0.05753 (13)	0.59407 (11)	0.0307 (3)	
C1	0.3305 (2)	0.58452 (17)	0.87724 (14)	0.0236 (3)	
C2	0.2441 (2)	0.53951 (17)	0.78490 (14)	0.0217 (3)	
H2	0.1206	0.6131	0.7772	0.026*	
C3	0.4071 (2)	0.56388 (18)	0.67298 (14)	0.0241 (3)	
H3A	0.4742	0.4670	0.6448	0.029*	
H3B	0.3475	0.6268	0.6112	0.029*	
C4	0.5553 (2)	0.64167 (17)	0.70584 (14)	0.0229 (3)	
C5	0.1782 (2)	0.38204 (17)	0.82204 (13)	0.0198 (3)	
H5	0.1048	0.3789	0.9058	0.024*	
C6	0.0298 (2)	0.35351 (18)	0.75498 (14)	0.0228 (3)	
H6A	−0.0792	0.4403	0.7555	0.027*	
H6B	−0.0331	0.2643	0.7946	0.027*	
C7	0.1347 (2)	0.32835 (17)	0.62825 (14)	0.0209 (3)	
H7	0.1755	0.4237	0.5823	0.025*	
C8	−0.0087 (2)	0.26947 (18)	0.57545 (14)	0.0235 (3)	
C9	0.2381 (2)	0.07047 (18)	0.59505 (13)	0.0226 (3)	
C10	0.3176 (2)	0.20368 (16)	0.61997 (13)	0.0194 (3)	
H10	0.4219	0.2337	0.5491	0.023*	
C11	0.4192 (2)	0.17137 (16)	0.72211 (13)	0.0183 (3)	
C12	0.3571 (2)	0.25765 (16)	0.81480 (13)	0.0190 (3)	
C13	0.4641 (2)	0.22721 (17)	0.90297 (13)	0.0222 (3)	
H13	0.4250	0.2865	0.9658	0.027*	
C14	0.6257 (2)	0.11244 (17)	0.90047 (14)	0.0237 (3)	
H14	0.6960	0.0934	0.9614	0.028*	
C15	0.6854 (2)	0.02504 (17)	0.80893 (14)	0.0213 (3)	
C16	0.5840 (2)	0.05678 (17)	0.71949 (13)	0.0206 (3)	
H16	0.6274	−0.0003	0.6554	0.025*	
C17	0.8514 (2)	−0.10779 (18)	0.80455 (15)	0.0259 (3)	
H17A	0.9082	−0.1267	0.7225	0.031*	
H17B	0.7930	−0.1975	0.8474	0.031*	

### Atomic displacement parameters (Å^2^)

**Table:**

	*U*^11^	*U*^22^	*U*^33^	*U*^12^	*U*^13^	*U*^23^
Cl1	0.0321 (2)	0.0391 (3)	0.0504 (3)	0.00849 (18)	−0.0251 (2)	−0.0182 (2)
O1	0.0324 (6)	0.0352 (7)	0.0283 (6)	−0.0042 (5)	−0.0050 (5)	−0.0130 (5)
O2	0.0287 (6)	0.0309 (6)	0.0285 (6)	−0.0087 (5)	−0.0100 (5)	−0.0079 (5)
O3	0.0303 (6)	0.0306 (6)	0.0324 (7)	−0.0097 (5)	−0.0082 (5)	−0.0032 (5)
O4	0.0269 (6)	0.0288 (6)	0.0394 (7)	−0.0020 (5)	−0.0181 (5)	−0.0038 (5)
O5	0.0256 (6)	0.0227 (6)	0.0339 (6)	−0.0028 (4)	−0.0137 (5)	−0.0070 (5)
O6	0.0334 (6)	0.0226 (6)	0.0396 (7)	0.0017 (5)	−0.0136 (5)	−0.0118 (5)
C1	0.0232 (7)	0.0202 (7)	0.0292 (8)	0.0000 (6)	−0.0082 (6)	−0.0084 (6)
C2	0.0205 (7)	0.0191 (7)	0.0277 (8)	0.0003 (6)	−0.0092 (6)	−0.0072 (6)
C3	0.0286 (8)	0.0213 (7)	0.0256 (8)	−0.0070 (6)	−0.0101 (6)	−0.0025 (6)
C4	0.0249 (8)	0.0184 (7)	0.0271 (8)	−0.0007 (6)	−0.0105 (6)	−0.0031 (6)
C5	0.0172 (7)	0.0202 (7)	0.0224 (7)	−0.0011 (5)	−0.0041 (6)	−0.0062 (6)
C6	0.0180 (7)	0.0226 (7)	0.0293 (8)	−0.0020 (6)	−0.0062 (6)	−0.0074 (6)
C7	0.0199 (7)	0.0183 (7)	0.0269 (8)	−0.0023 (5)	−0.0096 (6)	−0.0032 (6)
C8	0.0241 (8)	0.0225 (7)	0.0257 (8)	−0.0041 (6)	−0.0088 (6)	−0.0028 (6)
C9	0.0237 (7)	0.0238 (8)	0.0220 (7)	−0.0023 (6)	−0.0075 (6)	−0.0055 (6)
C10	0.0190 (7)	0.0198 (7)	0.0204 (7)	−0.0026 (5)	−0.0059 (5)	−0.0035 (6)
C11	0.0182 (7)	0.0174 (7)	0.0202 (7)	−0.0046 (5)	−0.0057 (5)	−0.0009 (5)
C12	0.0184 (7)	0.0177 (7)	0.0214 (7)	−0.0036 (5)	−0.0049 (5)	−0.0018 (5)
C13	0.0266 (8)	0.0203 (7)	0.0207 (7)	−0.0045 (6)	−0.0059 (6)	−0.0040 (6)
C14	0.0261 (8)	0.0223 (7)	0.0253 (8)	−0.0038 (6)	−0.0116 (6)	−0.0010 (6)
C15	0.0202 (7)	0.0183 (7)	0.0263 (8)	−0.0027 (5)	−0.0076 (6)	−0.0015 (6)
C16	0.0209 (7)	0.0191 (7)	0.0229 (7)	−0.0028 (6)	−0.0054 (6)	−0.0054 (6)
C17	0.0254 (8)	0.0209 (7)	0.0345 (9)	−0.0002 (6)	−0.0137 (7)	−0.0050 (6)

### Geometric parameters (Å, º)

**Table:**

Cl1—C17	1.7920 (16)	C6—H6A	0.9900
O1—C1	1.187 (2)	C6—H6B	0.9900
O2—C4	1.384 (2)	C7—C8	1.510 (2)
O2—C1	1.393 (2)	C7—C10	1.535 (2)
O3—C4	1.192 (2)	C7—H7	1.0000
O4—C8	1.1957 (19)	C9—C10	1.520 (2)
O5—C8	1.3818 (19)	C10—C11	1.520 (2)
O5—C9	1.3923 (19)	C10—H10	1.0000
O6—C9	1.189 (2)	C11—C12	1.395 (2)
C1—C2	1.518 (2)	C11—C16	1.398 (2)
C2—C3	1.529 (2)	C12—C13	1.399 (2)
C2—C5	1.552 (2)	C13—C14	1.384 (2)
C2—H2	1.0000	C13—H13	0.9500
C3—C4	1.503 (2)	C14—C15	1.390 (2)
C3—H3A	0.9900	C14—H14	0.9500
C3—H3B	0.9900	C15—C16	1.386 (2)
C5—C12	1.515 (2)	C15—C17	1.509 (2)
C5—C6	1.528 (2)	C16—H16	0.9500
C5—H5	1.0000	C17—H17A	0.9900
C6—C7	1.538 (2)	C17—H17B	0.9900
			
C4—O2—C1	110.80 (12)	C6—C7—H7	110.6
C8—O5—C9	110.77 (12)	O4—C8—O5	120.53 (14)
O1—C1—O2	120.33 (14)	O4—C8—C7	129.30 (15)
O1—C1—C2	129.76 (15)	O5—C8—C7	110.11 (13)
O2—C1—C2	109.90 (13)	O6—C9—O5	120.05 (15)
C1—C2—C3	103.52 (12)	O6—C9—C10	130.51 (15)
C1—C2—C5	110.96 (13)	O5—C9—C10	109.38 (13)
C3—C2—C5	118.62 (12)	C9—C10—C11	114.41 (12)
C1—C2—H2	107.8	C9—C10—C7	103.68 (12)
C3—C2—H2	107.8	C11—C10—C7	116.64 (12)
C5—C2—H2	107.8	C9—C10—H10	107.2
C4—C3—C2	105.08 (13)	C11—C10—H10	107.2
C4—C3—H3A	110.7	C7—C10—H10	107.2
C2—C3—H3A	110.7	C12—C11—C16	119.82 (13)
C4—C3—H3B	110.7	C12—C11—C10	121.42 (13)
C2—C3—H3B	110.7	C16—C11—C10	118.70 (13)
H3A—C3—H3B	108.8	C11—C12—C13	118.55 (14)
O3—C4—O2	120.22 (14)	C11—C12—C5	121.75 (13)
O3—C4—C3	129.78 (15)	C13—C12—C5	119.70 (13)
O2—C4—C3	109.95 (13)	C14—C13—C12	121.30 (14)
C12—C5—C6	110.91 (12)	C14—C13—H13	119.3
C12—C5—C2	112.48 (12)	C12—C13—H13	119.3
C6—C5—C2	112.64 (13)	C13—C14—C15	120.07 (14)
C12—C5—H5	106.8	C13—C14—H14	120.0
C6—C5—H5	106.8	C15—C14—H14	120.0
C2—C5—H5	106.8	C16—C15—C14	119.13 (14)
C5—C6—C7	111.94 (12)	C16—C15—C17	117.99 (14)
C5—C6—H6A	109.2	C14—C15—C17	122.83 (14)
C7—C6—H6A	109.2	C15—C16—C11	121.09 (14)
C5—C6—H6B	109.2	C15—C16—H16	119.5
C7—C6—H6B	109.2	C11—C16—H16	119.5
H6A—C6—H6B	107.9	C15—C17—Cl1	112.50 (11)
C8—C7—C10	103.64 (12)	C15—C17—H17A	109.1
C8—C7—C6	108.82 (13)	Cl1—C17—H17A	109.1
C10—C7—C6	112.36 (12)	C15—C17—H17B	109.1
C8—C7—H7	110.6	Cl1—C17—H17B	109.1
C10—C7—H7	110.6	H17A—C17—H17B	107.8
			
C4—O2—C1—O1	176.48 (15)	O6—C9—C10—C7	−170.26 (17)
C4—O2—C1—C2	−4.14 (16)	O5—C9—C10—C7	12.71 (16)
O1—C1—C2—C3	−172.78 (17)	C8—C7—C10—C9	−15.08 (15)
O2—C1—C2—C3	7.90 (16)	C6—C7—C10—C9	102.23 (14)
O1—C1—C2—C5	−44.5 (2)	C8—C7—C10—C11	−141.81 (13)
O2—C1—C2—C5	136.19 (13)	C6—C7—C10—C11	−24.50 (18)
C1—C2—C3—C4	−8.38 (15)	C9—C10—C11—C12	−125.35 (15)
C5—C2—C3—C4	−131.77 (13)	C7—C10—C11—C12	−4.1 (2)
C1—O2—C4—O3	−179.38 (14)	C9—C10—C11—C16	57.57 (18)
C1—O2—C4—C3	−1.65 (17)	C7—C10—C11—C16	178.79 (13)
C2—C3—C4—O3	−175.99 (16)	C16—C11—C12—C13	0.5 (2)
C2—C3—C4—O2	6.56 (17)	C10—C11—C12—C13	−176.56 (13)
C1—C2—C5—C12	−72.89 (16)	C16—C11—C12—C5	−179.44 (13)
C3—C2—C5—C12	46.72 (18)	C10—C11—C12—C5	3.5 (2)
C1—C2—C5—C6	160.87 (12)	C6—C5—C12—C11	25.70 (19)
C3—C2—C5—C6	−79.51 (16)	C2—C5—C12—C11	−101.46 (16)
C12—C5—C6—C7	−54.14 (17)	C6—C5—C12—C13	−154.22 (14)
C2—C5—C6—C7	72.94 (16)	C2—C5—C12—C13	78.62 (17)
C5—C6—C7—C8	168.26 (12)	C11—C12—C13—C14	−1.2 (2)
C5—C6—C7—C10	54.08 (17)	C5—C12—C13—C14	178.68 (14)
C9—O5—C8—O4	176.79 (15)	C12—C13—C14—C15	0.3 (2)
C9—O5—C8—C7	−5.78 (17)	C13—C14—C15—C16	1.5 (2)
C10—C7—C8—O4	−169.45 (17)	C13—C14—C15—C17	−176.01 (14)
C6—C7—C8—O4	70.8 (2)	C14—C15—C16—C11	−2.3 (2)
C10—C7—C8—O5	13.41 (16)	C17—C15—C16—C11	175.37 (14)
C6—C7—C8—O5	−106.35 (14)	C12—C11—C16—C15	1.3 (2)
C8—O5—C9—O6	177.93 (15)	C10—C11—C16—C15	178.39 (14)
C8—O5—C9—C10	−4.67 (17)	C16—C15—C17—Cl1	147.27 (13)
O6—C9—C10—C11	−42.1 (2)	C14—C15—C17—Cl1	−35.2 (2)
O5—C9—C10—C11	140.82 (13)		

### Hydrogen-bond geometry (Å, º)

**Table:**

*D*—H···*A*	*D*—H	H···*A*	*D*···*A*	*D*—H···*A*
C3—H3*B*···O4^i^	0.99	2.51	3.420 (2)	153
C5—H5···O1^ii^	1.00	2.59	3.386 (2)	136
C7—H7···O4^i^	1.00	2.51	3.468 (2)	160
C14—H14···Cl1	0.95	2.75	3.1045 (17)	103

Symmetry codes: (i) −*x*, −*y*+1, −*z*+1; (ii) −*x*, −*y*+1, −*z*+2.
